# Design of TAT-Conjugated Bowman–Birk Trypsin Inhibitor Peptides with Enhanced Antimicrobial and Antiproliferative Activities

**DOI:** 10.3390/biom16040511

**Published:** 2026-03-30

**Authors:** Ying Wang, Yangyang Jiang, Tao Wang, Xiaoling Chen, Lei Wang, Mei Zhou, James F. Burrows, Tianbao Chen, Xiaofei Zhang, Na Li

**Affiliations:** 1 Shaanxi Province Key Laboratory of New Drugs and Chinese Medicine Foundation Research, School of Pharmacy, Shaanxi University of Chinese Medicine, Xi’an 712046, China; wangying011@sntcm.edu.cn; 2 Natural Drug Discovery Group, School of Pharmacy, Queen’s University Belfast, Belfast BT9 7BL, UK; yangyangjiang@qub.ac.uk (Y.J.); t.wang@qub.ac.uk (T.W.); x.chen@qub.ac.uk (X.C.); l.wang@qub.ac.uk (L.W.); m.zhou@qub.ac.uk (M.Z.); j.burrows@qub.ac.uk (J.F.B.); t.chen@qub.ac.uk (T.C.)

**Keywords:** Bowman–Birk type inhibitors, the transactivator of transcription (TAT) peptide, linker, antimicrobial activity, antiproliferative activity

## Abstract

Cell-penetrating peptide (CPP) conjugation represents a promising strategy for enhancing the biological activity of therapeutic peptides. In this study, three analogues were designed by conjugating the trypsin inhibitory loop (TIL) derived from a Bowman–Birk-type inhibitor with the transactivator of transcription (TAT) peptide to improve their bioactivity. All TAT-TIL conjugates exhibited significantly enhanced antimicrobial activity compared with the parent peptide. Notably, the analogue containing a glycine linker (-GG-) showed further improvement in antiproliferative activity against cancer cells, indicating the potential role of linker design in optimizing peptide function. All analogues exhibited low hemolytic activity at the highest tested concentrations, although increased cytotoxicity toward normal HaCaT cells was observed, suggesting the need for further optimization of selectivity. Interestingly, comparable antimicrobial activities were observed regardless of protease inhibitory capacity, indicating that protease inhibition is not essential for the enhanced biological effects. Overall, TAT conjugation significantly improves the biological activity of Bowman–Birk-type inhibitor-derived peptides, and the incorporation of a glycine linker further enhances their functional properties. These findings support CPP-mediated peptide modification as an effective strategy for developing potential antimicrobial and anticancer peptide candidates.

## 1. Introduction

Cell-penetrating peptides (CPPs) exhibit diversity in size, amino acid sequences, and charge profile; however, they are typically short peptides comprising 5 to 30 amino acids. A common characteristic among all CPPs is their capability to traverse the plasma membrane, specifically or non-specifically, thereby facilitating the delivery of diverse molecular cargoes, including various bioactive substances, either to the cytoplasm or specific organelles. Cell-penetrating peptides can enhance the cellular absorption and uptake of a diverse range of molecules, extending from nanoscale particles to minor chemical entities and large DNA segments. The association of this “cargo” with the peptides occurs either through covalent bond-based chemical linkages or via non-covalent interactions [1,2,3,4]. The numerous applications of CPPs in medicine include roles as drug delivery agents for treating various diseases, such as cancer and viral infections, and as contrast agents for cell labelling. Important examples of the latter include the fact that they serve as carriers for Green Fluorescent Protein (GFP), MRI contrast agents, and quantum dots [5]. CPPs are categorized as polycationic, amphipathic and hydrophobic peptides based on their structural variations, which are characterized by an amino acid composition that predominantly includes positively charged amino acids like lysine or arginine, or they may present sequences featuring an alternating arrangement of polar, charged amino acids and non-polar, hydrophobic amino acids [6,7,8].

Cationic cell-penetrating peptides predominantly consist of a rich composition of arginine or lysine residues, each regularly bearing a minimum of five positive charges. The potent capacity of CPPs to deliver substances that inherently lack membrane penetration capabilities has been empirically validated. Among these, the TAT peptide, derived from the transactivator of transcription (TAT) protein of human immunodeficiency virus type 1 (HIV-1), is a well-known CPP [2,9]. The TAT sequence was identified and denoted as YGRKKRRQRRR, and its remarkable ability to transport substances into cells was demonstrated. This is attributed to its R/K-rich core sequence (RKKRRQRRR), with membrane interactions primarily governed by electrostatic charge [10,11,12]. Subsequently, a range of cationic CPPs sharing structural similarities with TAT, such as the presence of polyarginine, low-molecular-weight protamine, and penetrating capacities, have been extensively employed in research. These peptides have found wide application not only in studies related to inflammatory conditions but also in investigations into cancer therapy [3,13,14,15].

Protease inhibitors (PIs) are recognized for their crucial functions in both the development and therapy of human diseases like cancer, inflammation, and hemorrhage. This is attributed to their ability to suppress the catalytic activity of proteolytic enzymes [16]. Bowman–Birk-like trypsin inhibitors (BBIs) are a subgroup within the Serine protease inhibitors (Serpins) family. The structure of BBI peptides derived from amphibian skin secretions is characterized by a conserved disulfide-bridged loop comprising eleven amino acid residues (CWTP1SXPPXPC). Serpins are prevalent in nature and exert an effect on sustaining homeostasis across almost all organisms. They act as regulators in immune responses and have been identified as having clinically important roles in a range of diseases, including inflammation, thrombosis, and cancer [17,18,19]. Anticancer properties in the family of amphibian skin-derived BBIs have been reported [20]. Every stage in the cancer process is considered to be associated with abnormal expression of proteases, including proliferation, migration and invasion [21,22,23,24]. Proteasome inhibition, particularly of the 20S proteasome, promotes ROS accumulation, resulting in mitochondrial dysfunction and apoptosis in breast cancer cells. The black-eyed pea trypsin/chymotrypsin inhibitor (BTCI) exerts anticancer effects through this proteasome inhibition-dependent apoptotic pathway [25].

In contrast to major antimicrobial peptides (AMPs), BBIs are considered to be safe for oral administration, and their therapeutic effects in the oral treatment of multiple sclerosis have been verified in animal experiments [26]. Moreover, BBI peptides showed a low degree of cytotoxicity on normal cell lines [27]. However, BBI peptides exhibit limited membrane permeability. Like other peptides, BBIs also encounter challenges such as low bioavailability, rapid clearance, and sometimes poor solubility. These factors collectively are drawbacks for BBI peptides in their development as clinical drugs. To address these limitations, previous studies have explored structural modification of BBI peptides through conjugation with cell-penetrating peptides (CPPs) to enhance membrane permeability and improve biological activity [20,28]. However, these approaches have primarily focused on general CPP conjugation strategies, while the effects of linker design and the underlying membrane interaction mechanisms have not been systematically investigated. In the present study, we specifically examine the conjugation of TAT with a BBI-derived trypsin inhibitory loop (TIL) and further investigate the role of glycine linkers in modulating peptide conformation, membrane interaction, and biological activity.

Peptide conjugation has emerged as a promising strategy for peptide modification and therapeutic development in recent decades. This approach proves effective in enhancing the functional properties of peptides, improving drug stability, extending half-life, and achieving targeted delivery. Peptide-based drug conjugation involves linking peptides with chemical drugs, nanoparticles, polymers, and other peptides, offering a versatile strategy for the development of advanced therapeutic agents [29,30,31,32]. The proportion of peptide conjugates and the diversity of conjugated moieties have increased over time. Since 2010, conjugated peptides have accounted for approximately 30% of peptides entering clinical development [33]. Cell-penetrating peptides (CPPs) are concise peptide sequences without obvious antimicrobial activity, which exhibit the potential to transport therapeutic agents to diverse cells and tissues. This suggests that CPPs could potentially integrate into pharmaceutical formulations as essential components in the future [34,35,36]. CPPs are used as the targeted delivery of antibiotics for combating bacterial infections, which is a viable approach for eradicating intracellular bacteria, or to develop chimeric peptides with antimicrobial activity [37,38,39].

In previous research, a BBI peptide, OSTI-1872, from *Odorrana schmackeri* (Genbank accession number: OR902190) was identified and displayed potent trypsin inhibitory activity and weak antimicrobial activity [40]. Three analogues were then designed by conjugating TAT to the TIL, thereby improving membrane permeability, and all the analogues exhibited significantly enhanced biological activities compared with the parent peptide. Interestingly, preliminary results suggested that the introduction of a glycine linker (-GG-) may further modulate peptide activity, particularly in relation to cancer cell proliferation. Based on these observations, the present study aims to systematically investigate the effects of CPP conjugation and linker design on the structure, membrane interaction, and biological activity of BBI-derived peptides. Specifically, we focus on TAT-TIL conjugates and examine the role of glycine linkers in modulating peptide conformation and membrane interaction behaviour. In addition, molecular dynamics simulations are employed to provide mechanistic insights into peptide–membrane interactions. This study, therefore, aims to elucidate how CPP conjugation and linker engineering collectively influence peptide function, rather than simply applying an established modification strategy.

## 2. Materials and Methods

### 2.1. Peptides Design and Solid Phase Peptides Synthesis (SPPS)

Three peptide analogues were rationally designed by conjugating the trypsin inhibitory loop (TIL) with the TAT peptide. One analogue was specifically designed to evaluate the effect of a short glycine linker (-GG-) on the biological activity of the conjugates. The parent peptide and its analogues were synthesized using an automated solid-phase peptide synthesizer (Protein Technologies, Tucson, AZ, USA). Each amino acid (0.3 mmol × 2.5 equivalents) was coupled using 2-(1H-benzotriazole-1-yl)-1,1,3,3-tetramethyluronium hexafluorophosphate (HBTU) as the coupling reagent. The synthesized peptides were cleaved from the resin using a cleavage mixture containing 94% trifluoroacetic acid (TFA), 2% double-distilled water (ddH_2_O), 2% thioanisole (TIS), and 2% 1,2-ethanedithiol (EDT). Peptides were synthesized using standard Fmoc-based solid-phase peptide synthesis (SPPS) on resins with a loading of ~0.3 mmol/g: MBHA resin for peptides with C-terminal amidation, or Wang resin for peptides with a free C-terminal carboxyl group. The filtrate was extracted with ether and centrifuged, followed by removal of the ether layer. The resulting product was air-dried, lyophilized, and stored at −20 °C.

### 2.2. Prediction of Physicochemical Properties and Secondary Structure

The peptides’ physicochemical properties were determined using the online proteomic bioinformatic resource BACHEM (https://www.bachem.com/knowledge-center/peptide-calculator/ (accessed on 10 July 2025)). Secondary structure prediction was performed using the online AlphaFold Server (https://alphafoldserver.com (accessed on 10 July 2025)).

### 2.3. Purification and Identification of Peptides

Crude peptides were purified by reverse-phase high-performance liquid chromatography (RP-HPLC) using a LUNA C-5 preparative column (250 × 10 mm, Phenomenex, Macclesfield, UK). The mobile phase consisted of solvent A (ddH_2_O containing 0.5% TFA) and solvent B (80% acetonitrile, 19.5% ddH_2_O, 0.5% TFA). Elution was performed using a linear gradient from 38% to 48% solvent B (corresponding to 62% to 52% solvent A) over 80 min at a flow rate of 5 mL/min, with UV detection at 214 nm. The target peptides were collected at retention times of 23–31 min.

Peptide identity was confirmed by matrix-assisted laser desorption–ionization time-of-flight mass spectrometry (MALDI-TOF MS) (Voyager DE, Perseptive Biosystems, Framingham, MA, USA). The matrix solution contained α-cyano-4-hydroxycinnamic acid (CHCA, 10 mg/mL) dissolved in 70% acetonitrile and 30% water with 0.1% TFA. HPLC fractions (2 μL) were mixed with 1 μL matrix solution, air-dried on the target plate, and analyzed to obtain mass-to-charge ratios (*m*/*z*).

### 2.4. Peptide Secondary Structural Analysis

The secondary structures were analyzed using a JASCO J815 circular dichroism (CD) spectrometer (Jasco, Essex, UK). Peptides were dissolved to a concentration of 100 μM with ammonium acetate (NH_4_Ac) solution (20 mM), acting as a stock solution. Then, the stock solution was mixed with an equal volume of ddH_2_O and TFE, respectively, to generate working solutions, which were used to mimic the aqueous environment and the membrane environment. The peptide working solutions were scanned at wavelengths ranging from 190 to 260 nm in a quartz cuvette with a thickness of 1 mm. The scanning speed was set at 200 nm/min, and the bandwidth and data pitch were 1 nm and 0.5 nm, respectively.

### 2.5. Trypsin/Chymotrypsin Inhibition Determinations

Trypsin/chymotrypsin inhibitory activity was evaluated [28] using Phe-Pro-Arg-AMC (Bachem, Saint Helens, UK) and Succinyl-Ala-Ala-Pro-Phe-AMC (Bachem, UK) as the substrate, respectively. AMC is the abbreviation of 7-amino-4-methylcoumarin. Peptides (1–1000 μM) were prepared in phosphate-buffered saline (PBS) and added to black 96-well plates containing 180 μL of substrate (50 μM) and 10 μL of trypsin working solution, yielding a final volume of 210 μL per well. The trypsin/chymotrypsin stock solution (1 mg/mL) was first diluted to 1:1000 with 1 mM HCl to prepare a working solution before use in the inhibition assay.

Fluorescence was measured immediately using a FLUOstar OPTIMA plate reader (BMG Labtech, Ortenberg, Germany) at 37 °C, with excitation and emission wavelengths of 395 nm and 460 nm, respectively. Measurements were recorded every 30 s for 30 min. Inhibition curves were analyzed using the Morrison equation in Prism 9.

Working conditions were trypsin substrate concentration [S] = 42.86 μM; enzyme concentration Et = 0.0020 μM; Km = 41.07 μM. For chymotrypsin, [S] = 42.86 μM, Et = 0.0020 μM, and Km = 17.15 μM.

### 2.6. Antimicrobial Assays

Antimicrobial activity was evaluated by determining the minimum inhibitory concentration (MIC) and minimum bactericidal concentration (MBC). Ten microorganisms were selected for use in this assay including *Escherichia coli* (*E. coli*, ATCC CRM 8739) (*E. coli*, BAA 2340) (*E. coli*, NCTC 13846), *Pseudomonas aeruginosa* (*P. aeruginosa*, ATCC CRM 9027), *Staphylococcus aureus* (*S. aureus*, ATCC CRM 6538), *Enterococcus faecium* (*E. faecium*, NCTC 12697), Methicillin-resistant *Staphylococcus aureus* (MRSA, NCTC 12493), *Klebsiella pneumonia* (*K. pneumonia*, ATCC CRM 43861), *Acinetobacter baumannii* (*A. baumannii*, BAA 747), and *Candida albicans* (*C. albicans*, ATCC 10231).

Bacteria were cultured in tryptic soy broth or nutrient broth at 37 °C, while fungi were cultured in a yeast extract peptone dextrose medium at 26 °C in an Orbital Shaker for 16–20 h at 120 rpm/min. Cultures were diluted to approximately 5 × 10^5^ CFU/mL. Peptides were dissolved in dimethyl sulfoxide (DMSO) (100–51,200 μM). Each well contained 99 μL bacterial suspension and 1 μL peptide solution. Norfloxacin (20 μg/mL, 62.6 μM) and amphotericin B (10 μg/mL, 10.8 μM) served as positive controls for bacteria and fungi, respectively. Plates were incubated for 20–24 h, and optical density was measured at 550 nm using a Synergy HT plate reader (Bio-Tek, Minneapolis, MN, USA), operating in endpoint measurement mode.

MIC was defined as the lowest concentration without visible growth. For MBC determination, samples from MIC assays were plated on solid media and incubated; the MBC values were determined by visual inspection of colony growth on agar plates after incubation and defined as the lowest peptide concentration showing no visible bacterial colonies.

### 2.7. Time-Killing Kinetic Assays

Time-killing kinetic assays were executed to explore the killing efficiency of peptides against bacteria. The concentrations of tested peptides were at values of 1× MIC, 2× MIC and 4× MIC, and the three strains tested in this assay were Escherichia coli (ATCC CRM 8739) and two drug-resistant strains of *Escherichia coli* (*E. coli*, NCTC 13846) (*E. coli*, BAA 2340). The bacteria were cultured using the same method as that employed in the antimicrobial activity determination assay. The bacterial culture was diluted to ×10^5^ CFU/mL after reaching the logarithmic growth phase. In total, 198 μL of the bacterial suspension and 2 μL of peptide solutions with concentrations at 1× MIC, 2× MIC and 4× MIC were added to separate sterile tubes, respectively. The bacterial-peptide mixture medium was diluted 10, 100 and 1000 times, and then 10 μL of the mixture medium at four different bacterial densities was inoculated onto plates with solid culture medium (NA) at the time points of 0, 5, 10, 15, 30, 60, 90, 120 and 180 min. All colonies were counted and recorded after the seeded plates were incubated at 37 °C for 24 h. The vehicle control was 198 μL bacteria medium treated with 2 μL DMSO, and the growth control was just 200 μL bacteria medium without any peptide solution treatments.

### 2.8. SYTOX^TM^ Green Permeability Assays

Membrane permeabilization was assessed using SYTOX™ green nucleic acid stain (Thermo Fisher Scientific, Waltham, MA, USA), which selectively penetrates cells with compromised membranes.

At first, *E. coli* (NCTC 13846/BAA 2340) was cultured in TSB medium in an Orbital Shaker at 37 °C overnight. After being subcultured for 2 h, the bacterial culture was centrifuged at 1000× *g* for 10 min, 4 °C, and then the culture medium was discarded. The bacteria at the bottom of the tube were washed gently twice with 5% TSB (in 0.85% NaCl). For the third time, the bacterial suspension was diluted with the same solution until its density reached the logarithmic growth phase, in which the OD value reached 0.70 at the detected wavelength of 590 nm. Subsequently, 40 μL peptide solutions at concentrations of 2.5× MIC, 5× MIC, and 10× MIC and 50 μL of bacterial suspension were loaded into a black 96-well plate, respectively, and then incubated for 2 h at 37 °C. Finally, 10 μL of SYTOX^TM^ green nucleic acid stain (5 μM) was added to the plate, and the mixture was incubated at 37 °C under dark conditions for another 5 min. The total reaction volume was 100 μL, resulting in final peptide concentrations of MIC, 2× MIC, and 4× MIC, respectively. The intensity of fluorescence was detected by Synergy HT (BioTech, Winooski, VT, USA) and wavelengths of excitation and emission were set at 485 and 528 nm, respectively. A bacterial medium was added with 5% TSB and acted as the negative control; the bacterial medium treated with Melittin peptide solution (8 μM) was set as the positive control; and 5% TSB alone was the blank control.

### 2.9. MD Simulations of Peptide–Anionic Lipid Membrane Interactions

Molecular dynamics (MD) simulations were performed for the parent peptide and its analogues to investigate their interactions with a model bacterial membrane. The membrane system was constructed using CHARMM-GUI and consisted of phosphatidylethanolamine (POPE) and phosphatidylglycerol (POPG) lipids at a molar ratio of 3:1. The membrane patch measured approximately 10.0 × 10.0 nm^2^ and contained 252 POPE and 84 POPG molecules. All peptide C-termini were amidated.

Predicted peptide structures were visualized and inspected using PyMOL 2.5.5, and the highest-scoring model for each peptide was selected for simulation. Each peptide was initially positioned above the membrane surface. The systems were solvated with water molecules, and Na^+^ and Cl^−^ ions were added to neutralize the net charge and achieve a physiological ionic strength of 0.15 M. The final systems contained 121,928, 126,056, and 127,391 atoms, respectively.

The CHARMM36 force field was used to parameterize both peptides and lipid components, while the TIP3P model was applied for water molecules. Force-field parameters were generated using the pdb2gmx module in GROMACS 2023.3. Following energy minimization, systems were equilibrated under NVT and NPT ensembles prior to 500 ns production MD simulations. Electrostatic interactions were calculated using the particle mesh Ewald method. Simulations were performed with a 2 fs time step, and coordinates were recorded every 10 ps. Periodic boundary conditions were applied throughout the simulations. Trajectory analysis and visualization were conducted using VMD 1.9.4.

### 2.10. Salt Ions and Serum Sensitivity

The sensitivity of the peptides to salt ions and serum was examined in the antimicrobial activity determination assay under salt ion and serum conditions. *E. coli* (ATCC CRM 8739) was the tested bacterium. Different concentrations of salts (150 mM NaCl, 4.5 mM KCl, 6 µM NH_4_Cl, 1 mM MgCl_2_, 2.5 mM CaCl_2_, and 4 mM FeCl_3_) were used to determine the influence of cationic substances in the bacterial culture on the antimicrobial activities of the peptides.

### 2.11. Haemolysis Assays

The purpose of the hemolysis assay is to evaluate the cytotoxicity of peptides against horse red blood cells in vitro. Erythrocytes were obtained from defibrinated horse blood and then washed with PBS to reach 4% suspension in PBS as a working solution. The peptide was dissolved in DMSO to prepare a stock solution, and then it was diluted in PBS to working concentrations ranging from 32 μM to 512 μM (DMSO was less than 1% in total volume). Then, 100 μL of different peptide concentration solutions and 100 μL of 4% erythrocyte suspension were mixed in tubes, respectively, and the tubes were placed in an incubator at 37 °C for 2 h. Afterwards, the erythrocyte suspensions were centrifuged for 10 min at 930× *g*, and the supernatant in each tube was carefully transferred into a 96-well plate. The absorbance associated with erythrocyte lysis was measured at 470 nm using the reader Synergy HT plate reader (BioTek, Shoreline, WA, USA). The positive control was 4% erythrocyte suspension treated with 1% Triton X-100 (Sigma-Aldrich, St. Louis, MO, USA), and the negative control contained 4% erythrocyte suspension and 1% DMSO solution (diluted by PBS).

### 2.12. Antiproliferation Assays

The antiproliferative activity of peptides against human cell lines was evaluated using the MTT assay, which is based on the conversion of MTT into water-insoluble formazan crystals using metabolically active cells. The tested cell lines included human breast cancer cells (MCF-7), human lung carcinoma cells (H838), human colorectal carcinoma cells (HCT116), and human glioblastoma astrocytoma cells (U251MG). Cells were treated with peptides at concentrations ranging from 10^−9^ to 10^−4^ M to assess anticancer activity. A human skin keratinocyte cell line (HaCaT) was used to evaluate cytotoxicity toward normal cells. All cell lines were obtained from the American Type Culture Collection (ATCC, Manassas, VA, USA).

Cells were cultured in 75 mL flasks containing 10 mL of the appropriate growth medium at 37 °C for 3–5 days. After removing the culture medium, cells were washed twice with PBS and detached using 3–4 mL trypsin for 2–3 min. The digestion was terminated by adding 8–10 mL fetal bovine serum (FBS). The cell suspension was centrifuged, the supernatant was discarded, and the pellet was resuspended in 4 mL complete growth medium to prepare a stock suspension. Cell density was determined using trypan blue staining and adjusted with FBS to the required densities (H838 and MCF-7: 8 × 10^4^ cells/mL; U251MG: 5 × 10^4^ cells/mL; HCT116 and HaCaT: 2 × 10^5^ cells/mL). Cells were seeded into 96-well plates at 100 μL per well.

After incubation for 20–24 h, the medium was replaced with serum-free medium for 4 h to induce cell starvation. Peptides were dissolved in DMSO (10^−2^ M stock) and diluted with serum-free medium to final concentrations of 10^−9^–10^−4^ M. Cells were then treated with 100 μL peptide solutions. The vehicle control consisted of 1 μL DMSO in 99 μL serum-free medium, while blank and growth controls contained serum-free medium only. After 24 h of treatment, a 10 μL MTT solution was added to each well and incubated for 2 h. The medium was removed, and formazan crystals were dissolved in 100 μL DMSO. After shaking for 10 min, absorbance was measured at 570 nm using a microplate reader (BioTek, Shoreline, WA, USA).

### 2.13. Apoptosis Detection Assays

Cell apoptosis was evaluated using Annexin V and propidium iodide (PI) dual staining, which distinguishes apoptotic and necrotic cells, followed by flow cytometry analysis. Human lung carcinoma H838 cells were used in this assay. Apoptosis was assessed using the Muse™ Annexin V and Dead Cell Reagent (EMD Millipore, Billerica, MA, USA) according to the manufacturer’s instructions.

H838 cells were cultured in 15 mL flasks, as described in the antiproliferation assay. When cell confluence reached approximately 80%, the culture medium was removed, and cells were washed twice with PBS. Cells were detached using 3 mL of EBSS/trypsin solution for 3 min, and digestion was terminated by adding fetal bovine serum (FBS). The cell suspension was centrifuged at 300× *g* for 7 min at 4 °C, and the pellet was resuspended in complete growth medium. Cells were then seeded into 24-well plates at an approximate density of 1 × 10^6^ cells/mL (1 mL per well) and incubated at 37 °C under 5% CO_2_ for 24 h.

After incubation, the medium was replaced with serum-free medium for 12 h to induce cell starvation. Cells were subsequently treated with peptide solutions at IC_10_, IC_50_, and IC_90_ concentrations for 6 h. IC_10_ and IC_90_ values were calculated from IC_50_ values using an online calculator GraphPad QuickCalcs (https://www.graphpad.com/quickcalcs/Ecanything1/, (accessed 15 March 2024)). Cells treated with 200 μM cisplatin, which served as the positive control. 

Following treatment, cells were detached using EBSS/trypsin, collected into 1.5 mL tubes, and centrifuged at 300× *g* for 7 min. The cell pellet was resuspended in PBS to a final concentration of 1 × 10^6^ cells/mL. Subsequently, 100 μL of cell suspension was stained with 100 μL Muse Annexin V and Dead Cell Reagent and incubated for at least 20 min at room temperature in the dark. Samples were analyzed using a Muse Cell Analyzer (EMD Millipore, Billerica, MA, USA).

### 2.14. Statistical Analysis

Statistical analysis of biological activity determination assays was conducted using software Prism 9 (GraphPad Software, Boston, MA, USA). One-way/two-way ANOVA was used to analyze the statistical significance of the difference. The data points are the mean of the independent experiments, and the error bar represents the standard error of the mean (SEM). Ns represents a non-significant difference; * is *p* < 0.5; ** is 0.001 < *p* < 0.01; *** is 0.0001 < *p* < 0.001; and **** is *p* < 0.0001.

## 3. Results

### 3.1. Physicochemical Properties and Secondary Structure Analysis

**Figure 1:**
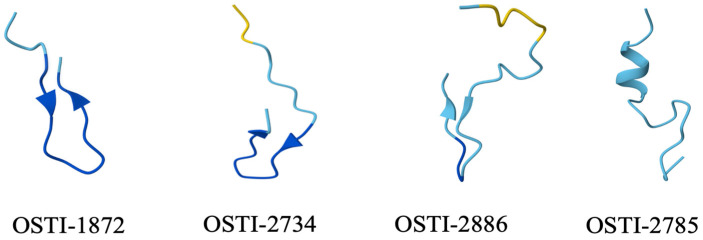
Predicted secondary structures of the peptides generated using the AlphaFold server. Blue indicates regions with pLDDT > 90; cyan represents regions with 70 < pLDDT ≤ 90; and yellow implies regions with 70 > plDDT > 50. The pLDDT score is an atom-level confidence metric ranging from 0 to 100, with higher values indicating greater reliability of the structural prediction.

The parent peptide, OSTI-1872, a novel Bowman–Birk-type trypsin inhibitor, was previously identified from the skin secretion of *Odorrana schmackeri* [40]. Based on its structural characteristics and amino acid sequence, three analogues were designed by conjugating the transactivator of the transcription (TAT) peptide with the trypsin inhibitory loop (TIL). OSTI-2734 was generated by directly fusing TAT with the TIL of OSTI-1872. In OSTI-2886, a di-glycine (-GG-) spacer was introduced between TAT and TIL to facilitate the linkage and improve structural flexibility. The final analogue, OSTI-2785, was derived from OSTI-2886 by disrupting the disulfide bridge through deletion of the terminal cysteine residue. After synthesizing the purified OSTI-1872 and its analogues (Figure S1), their physicochemical properties were calculated using the BACHEM programme (Table 1). Their secondary structures were also predicted using the online AlphaFold Server (Figure 1).

### 3.2. Circular Dichroism (CD) Spectroscopy

**Figure 2:**
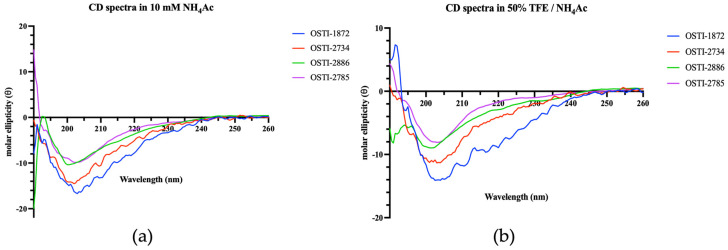
The CD spectra of peptides were detected in two different environments: (**a**) a 10 mM NH_4_Ac buffer, which acts as an aqueous environment, and (**b**) a 50% TFE/NH_4_Ac solution, which is a mimetic microbial membrane environment.

The secondary structures of the peptides were characterized by circular dichroism (CD) spectroscopy in an aqueous environment (10 mM ammonium acetate, NH_4_Ac) and a membrane-mimetic environment consisting of 50% (*v*/*v*) trifluoroethanol (TFE) in 10 mM NH_4_Ac (Figure 2). In aqueous solution, OSTI-1872 displayed moderate α-helical content, whereas the analogues, particularly OSTI-2785, showed increased β-strand content. Under membrane-mimetic conditions, OSTI-1872 exhibited a pronounced increase in α-helicity, while the analogues maintained or further increased β-strand conformations, indicating distinct structural responses to membrane-like environments. Overall, CD analysis demonstrated that the secondary structures of these peptides were strongly environment-dependent, with all peptides exhibiting mixed conformations of α-helix, β-strand, and random coil in both aqueous and membrane-mimetic conditions. These observations were consistent with the predicted structural results.

### 3.3. Trypsin/Chymotrypsin Inhibition Determinations

**Figure 3:**
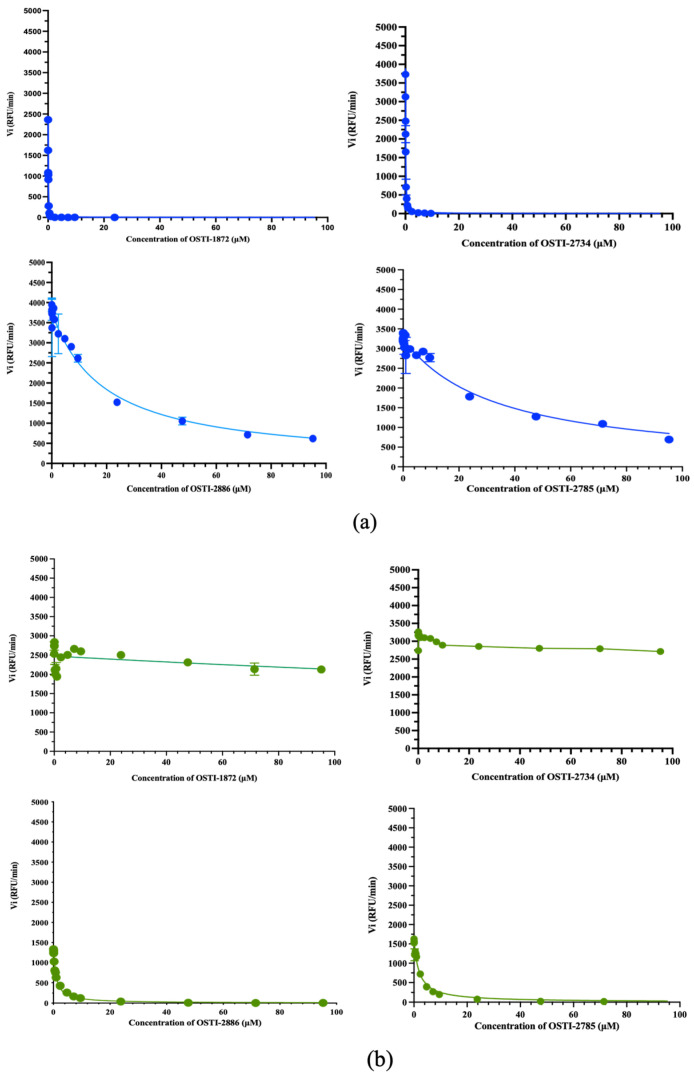
Progress curve for trypsin (**a**) and chymotrypsin (**b**) proteolysis in the presence of different concentrations of peptides. The data on concentration were converted to a logarithmic format. The statistical analysis was processed using the Morrison Ki programme; the error bar represents the mean + SEM (standard error of the mean) (*n* = 6).

The trypsin and chymotrypsin inhibitory activities of the parent peptide and analogues were evaluated using substrate hydrolysis progress curves (Table 2). The Morrison inhibition plots of the tested peptides are shown in Figure 3. All four peptides exhibited potent trypsin inhibitory activity when lysine occupied the P1 position, consistent with the inhibitory profiles of other amphibian-derived Bowman–Birk-type trypsin inhibitors. Substitution of the P1 residue from lysine to phenylalanine shifted the inhibitory specificity from trypsin to chymotrypsin, as demonstrated by OSTI-2886 and OSTI-2785.

### 3.4. Antimicrobial Activity Determinations

The parent peptide showed moderate antimicrobial activity against Gram-negative bacterial strains of *E. coli,* including two drug-resistant strains: *E. coli* BAA 2340 and *E. coli* NCTC 13846. These analogues, particularly OSTI-2886 and OSTI-2785 with the linker, showed the greatest improvement in antimicrobial activity against Gram-negative bacteria such as *E. coli*, *P. aeruginosa*, and *K. pneumoniae* and Gram-positive bacteria such as MRSA, especially two drug-resistant bacterial strains of *E. coli* (Table 3).

### 3.5. Time-Killing Assay

**Figure 4:**
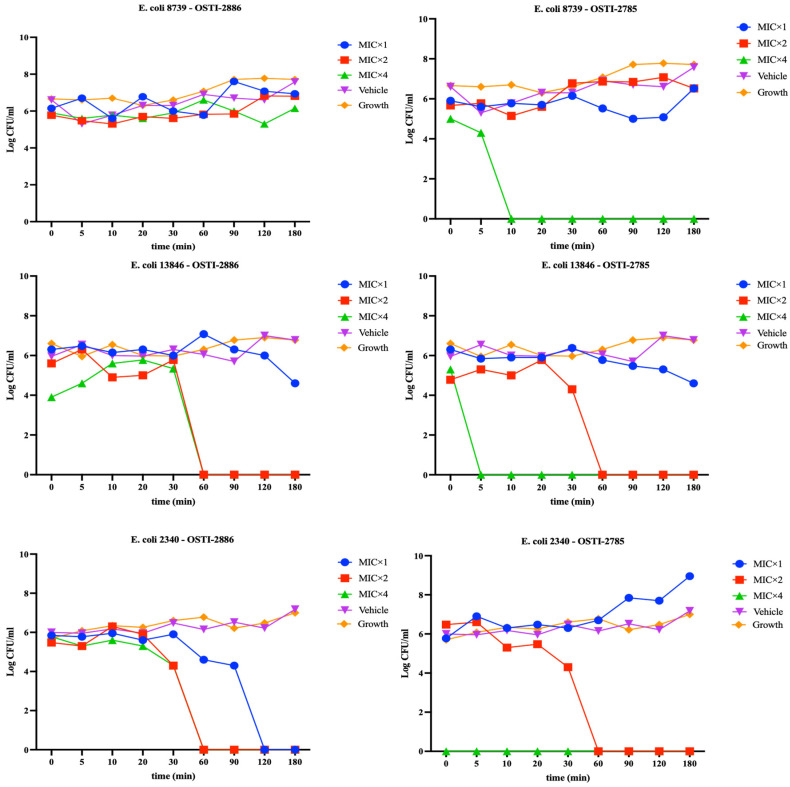
Killing kinetics of OSTI-2886 and OSTI-2785 against *E. coli* (ATCC CRM 8739), *E. coli* (NCTC 13846) and *E. coli* (BAA 2340). The bacteria were treated with peptides for 180 min at concentrations corresponding to 1× MIC, 2× MIC and 4× MIC. The bacteria treated with broth only were used as growth controls. The error bars represent the mean ± standard error of the mean (SEM).

Based on the MIC and MBC values, OSTI-2886 and OSTI-2785 were selected to assess their kinetic bactericidal efficacy at 1×, 2×, and 4× MIC. Overall, both analogues exhibited very limited bactericidal activity against the three selected bacterial strains at all tested concentrations. Their antibacterial effects against the two drug-resistant strains were only observed after more than 180 min. Notably, OSTI-2886 achieved complete bacterial killing within 120 min against *E. coli* (BAA-2340) at its MIC. However, increasing the concentration to 2× MIC resulted in only marginal improvement in bactericidal activity for both analogues (Figure 4).

### 3.6. SYTOX^TM^ Green Permeability Assays

**Figure 5:**
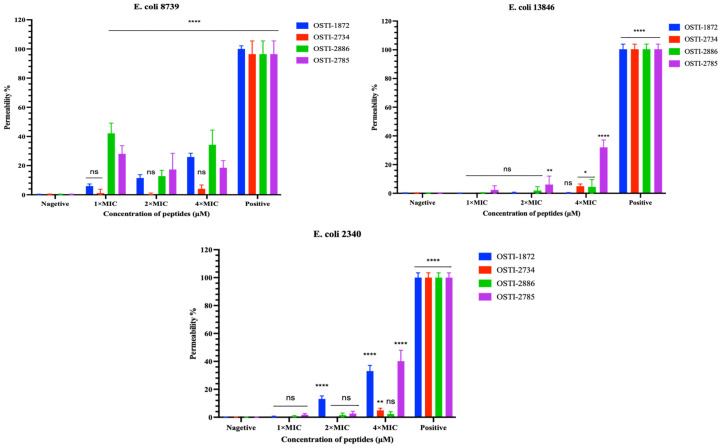
Cell membrane permeabilization effects of analogues against *E. coli* (ATCC CRM 8739), *E. coli* (NCTC 13846), and *E. coli* (BAA 2340) at concentrations of 1× MIC, 2× MIC and 4× MIC. Bacteria incubated in TSB were treated as the negative controls. Melittin (8 μM) was used as the positive control. The error bars represent the mean ± standard error of the mean (SEM). ns represents a non-significant difference; * is *p* < 0.5; ** is 0.001 < *p* < 0.01; and **** is *p* < 0.0001.

The SYTOX™ Green assay was conducted to investigate peptide-induced membrane permeability in *E. coli* strains. The results indicate that peptide-induced membrane permeabilization varies among bacterial strains (Figure 5). Generally, all four peptides produced no more than 40% membrane permeabilization across all tested bacteria, and it seems that non-drug-resistant *E. coli* strains exhibited greater peptide-induced membrane disruption than drug-resistant strains in this assay. The permeability induced by OSTI-2886 and OSTI-2785 against two drug-resistant strains increased in a concentration-dependent manner, showing the highest membrane-disruptive activity at 4× MIC. By contrast, OSTI-2734 exhibited the lowest permeability.

### 3.7. Salt Ions and Serum Assays

This assay aimed to study the stability of peptides against *E. coli* ATCC CRM 8739 under diverse salt ion and horse serum conditions. The results (Table 4) show that almost all peptides maintained a degree of stable antibacterial effects when treated with horse serum and salt ions, whereas CaCl_2_, NaCl, and MgCl_2_ exerted minor effects on the antibacterial activity of the parent peptide and its analogues.

### 3.8. MD Simulations

Molecular dynamics simulations were performed on structurally distinct peptides, including the parent peptide and its analogues, to compare their interactions with a simulated bacterial membrane.

#### 3.8.1. Representative Snapshots of the Molecular Dynamics Process

**Figure 6:**
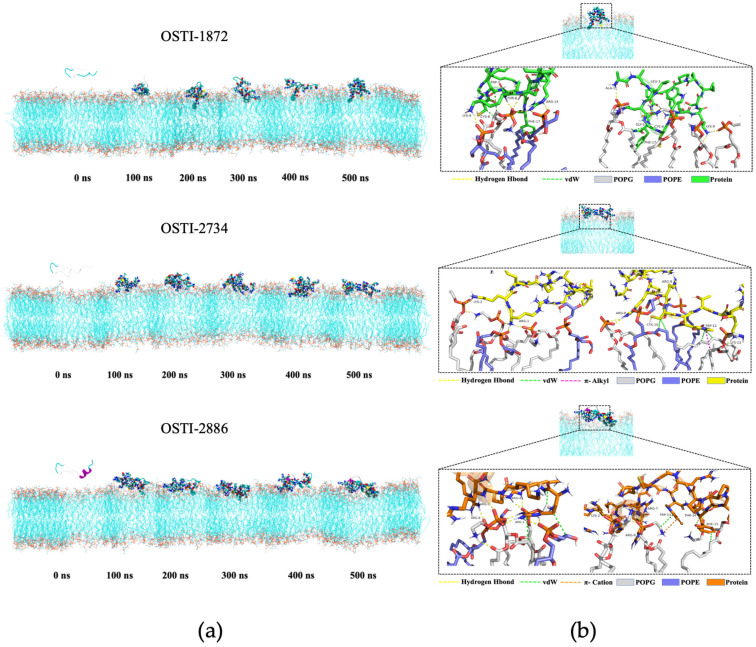
(**a**) Representative snapshots from molecular dynamics simulations: the peptide is shown in cartoon mode; the mixed cell membrane is shown in line mode; and the peptide amino acid residues within 4 Å of the cell membrane are shown in VDW mode for POPE, POPG, and water molecules. (**b**) Details of the interaction between the peptide and mixed cell membranes during molecular dynamics. In the figures, oxygen, nitrogen, and carbon atoms are represented in red, blue, and orange, respectively.

Figure 6 presents representative snapshots from the molecular dynamics simulations. During the simulations, all three peptides stably adsorbed onto the mixed POPE/POPG membrane, with several amino acid residues partially inserted into the lipid bilayer, indicating stable peptide–membrane interactions. However, the adsorption and insertion patterns varied among the peptides, suggesting distinct interaction modes with the mixed membrane.

#### 3.8.2. CSA and Contacts Analysis

**Figure 7:**
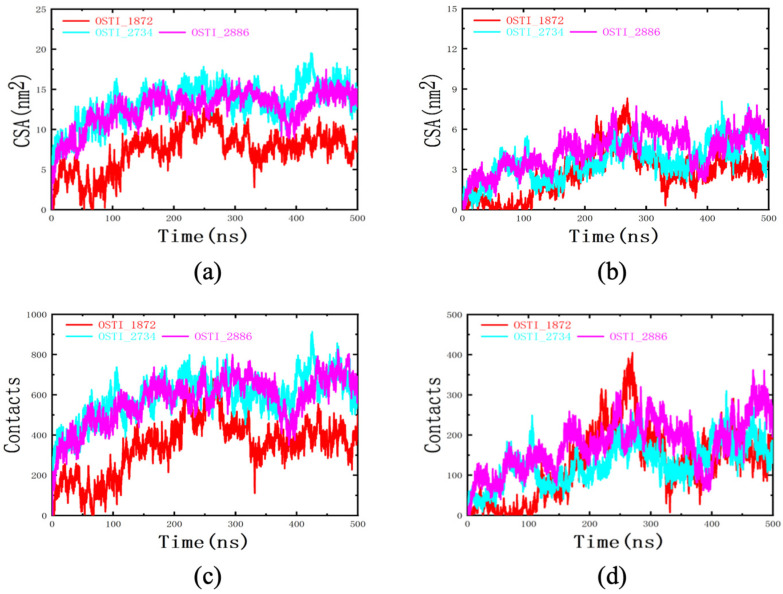
Contact analysis of peptides with the cell membrane and its hydrophobic core during molecular dynamics simulations. (**a**) Contact surface area (CSA) between each peptide and the mixed lipid membrane. (**b**) CSA between each peptide and the hydrophobic tail region of the membrane. (**c**) Number of contact atoms between each peptide and the mixed lipid membrane. (**d**) Number of contact atoms between each peptide and the hydrophobic tail region of the membrane.

Contact surface area (CSA) and contact atom number analyses were performed to quantify the extent of interaction between each peptide and the mixed lipid membrane, as well as between the peptides and the membrane tail region (Figure 7). The results showed that, compared with the parent peptide, the TAT-conjugated analogues exhibited larger contact surface areas and greater numbers of contact atoms with the cell membrane. Specifically, OSTI-1872 displayed a CSA of approximately 8 nm^2^ and interacted with around 410 membrane atoms, whereas OSTI-2734 and OSTI-2886 exhibited CSAs of roughly 14 nm^2^ and contacted about 630 membrane atoms.

#### 3.8.3. Centroid Distance Analysis

**Figure 8:**
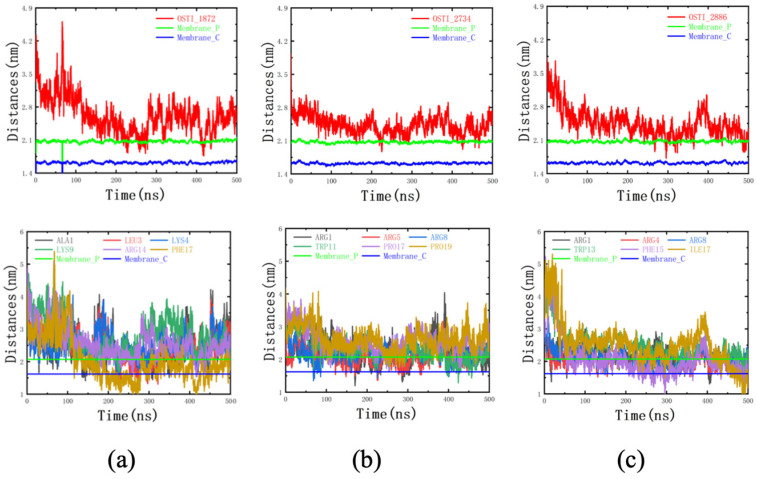
Centroid distance analysis between the three peptides and the mixed cell membrane during molecular dynamics simulations. The centroid distances between (**a**) OSTI-1872, (**b**) OSTI-2734, (**c**) OSTI-2886 and the membrane, as well as the centroid distances between the top three highest free binding energies with polar (charged) and non-polar residues and the mixed lipid membrane. Membrane_P denotes the plane defined by the phosphorus atoms of the phospholipid headgroups, and Membrane_C denotes the plane defined by the carbonyl carbon atoms of the phospholipid molecules.

The distance between each peptide and the centroid of the mixed membrane was predominantly maintained between 2.1 and 2.8 nm; it remained above the plane defined by the phospholipid phosphorus atoms (Figure 8), indicating that most peptide residues primarily interacted with the membrane surface. However, transient decreases in the peptide–membrane centroid distance to below 2.1 nm suggested partial insertion of certain residues into the membrane.

Notably, the non-polar residues of OSTI-1872 exhibited relatively deep membrane insertion, with centroid distances extending below the plane defined by the phospholipid carbonyl carbon atoms (~1.6 nm), enabling direct interactions with the hydrophobic acyl chains of the lipid bilayer. Similarly, OSTI-2886 occasionally displayed centroid distances of non-polar residues below the carbonyl carbon plane, indicating intermittent deep insertion into the hydrophobic core. In contrast, the non-polar residues of OSTI-2734 remained largely above the carbonyl carbon plane, suggesting that this peptide primarily interacted with the hydrophilic headgroup region and exhibited more limited penetration into the membrane interior.

#### 3.8.4. Analysis of Interaction Energy and Binding Free Energy

**Figure 9:**
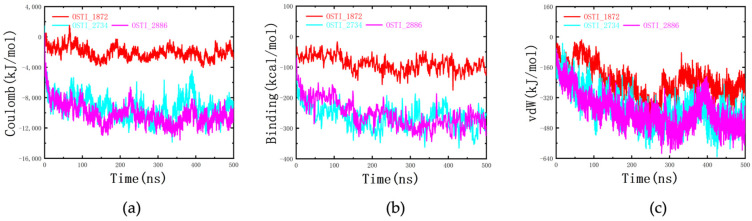
Energy analysis during the interaction process. (**a**) Electrostatic (Coulomb) interaction energy between each peptide and the cell membrane. (**b**) The binding free energy between each peptide and the cell membrane. (**c**) Van der Waals (vdW) interaction energy between each peptide and the cell membrane.

The van der Waals interaction energies between the peptides and the mixed cell membrane were on the order of several hundred kilojoules per mole, whereas the electrostatic interaction energies reached several thousand kilojoules per mole (Figure 9). This indicates that electrostatic forces dominate the interactions between the tested peptides and the membrane. Among the peptides, OSTI-2734 and OSTI-2886 exhibited stronger interactions with the mixed membrane, showing van der Waals energies of approximately −480 kJ/mol and electrostatic energies of roughly −9600 kJ/mol. In contrast, OSTI-1872 displayed much weaker interactions, with van der Waals and electrostatic energies of approximately −240 kJ/mol and −2000 kJ/mol, respectively. Binding free energies calculated using the MM/PBSA method followed the same trend. OSTI-2734 and OSTI-2886 exhibited the most favourable binding energies, both at around −280 kcal/mol, whereas the binding energy of OSTI-1872 was approximately −100 kcal/mol. Collectively, the interaction energy and binding free-energy analyses demonstrate that the TAT-conjugated analogues possess substantially enhanced membrane-binding affinity compared with the parent peptide.

### 3.9. MTT Cancer Cell Antiproliferation Assays

**Figure 10:**
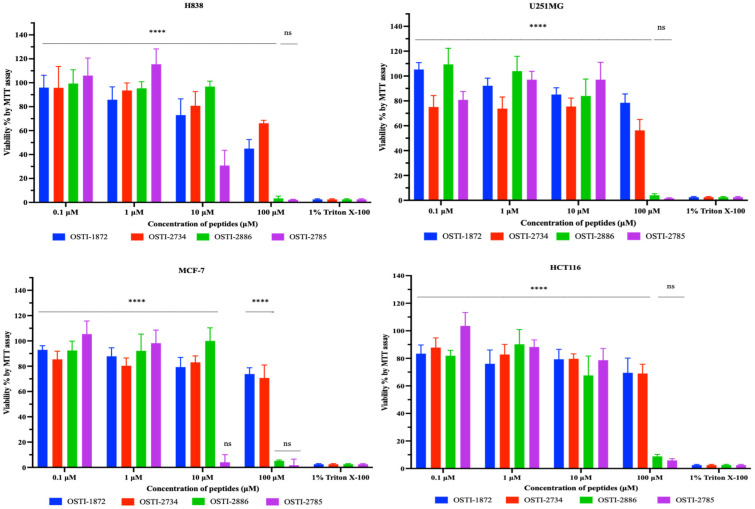
Cell viability of H838, U251MG, and HCT116 cells after 24 h of treatment with peptides. The cell viability of growth controls was regarded as 100%. Each concentration of the peptide is represented as the mean ± standard error of the mean (SEM) over nine replicates. ns represents a non-significant difference, and **** is *p* < 0.0001.

MTT assays were performed to examine the antiproliferation activities of the parent peptide and analogues (Figure 10); the IC_50_ values of peptides against the tested cell lines are shown in Table 5. In summary, the linker group (-GG-) appears to play a distinct and important role in the structural modification of analogues, compared with analogues without this linker. Those containing the linker exhibit markedly enhanced antiproliferative activity against cancer cells. OSTI-2886 and OSTI-2785 displayed a great improvement in antiproliferation activity against all tested cancer cell lines, in contrast to OSTI-2734, which showed no obvious anticancer activity.

### 3.10. Apoptosis Assays

**Figure 11:**
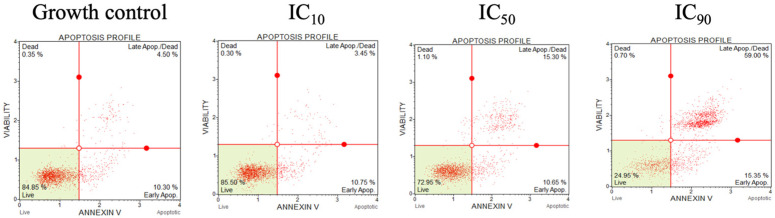
Flow cytometric analysis of Annexin V/PI staining. The prostate cancer cells H838 were treated with peptides for 6 h. Cells were dyed with Annexin V/PI and analyzed by flow cytometry. The cells treated with serum-free medium acted as growth controls.

Annexin V/propidium iodide (PI) apoptosis assays were performed to investigate the anticancer mechanism of OSTI-2886 by quantifying apoptosis in H838 cells after 6 h of treatment (Figure 11). Based on the IC_50_ value of 31.9 μM, the corresponding IC_10_ and IC_90_ values were calculated as 3.54 μM and 287.1 μM, respectively. The results showed that the apoptotic rate of H838 cells treated with OSTI-2886 increased only slightly across the tested concentrations, ranging from 10.75% to 15.35%. Compared with the control group (10.30%), these differences were not statistically significant, and no obvious increase in apoptosis was observed even at the IC90 concentration, suggesting that OSTI-2886 did not induce significant apoptosis in H838 cells under the experimental conditions.

### 3.11. Safety Evaluations

**Figure 12:**
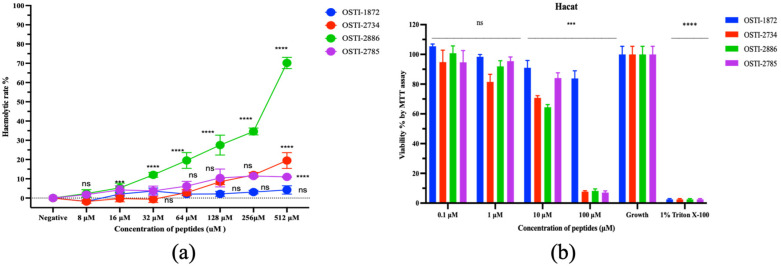
(**a**) Haemolytic activity of peptides to horse erythrocytes. (**b**) Cell viability of HaCaT cells after 24 h treatment with peptides. The cell viability of growth controls was regarded as 100%. Each concentration of peptide is represented as the mean ± standard error of the mean (SEM) over nine replicates. ns represents a non-significant difference; *** is 0.0001 < *p* < 0.001; and **** is *p* < 0.0001.

The haemolytic activity of the peptides toward horse erythrocytes (Figure 12a) and their antiproliferative effects on the normal HaCaT cell line (Figure 12b) were evaluated. Generally, the analogues exhibited increased haemolytic activity compared with the parent peptide, especially for OSTI-2886, which showed a marked increase when the peptide concentration was over 256 μM, with an HC_50_ value of 314.9 μM, while OSTI-2785 maintained a stable and low haemolytic activity after the last cysteine residue was removed and the disulfide bond was disrupted. Regarding antiproliferative activity, the parent peptide OSTI-1872 showed no significant effect on the proliferation of HaCaT cells. In contrast, the analogues OSTI-2886 and OSTI-2785 demonstrated significant antiproliferative effects on the HaCaT cell line at the highest tested concentration of 100 μM.

## 4. Discussion

Peptide conjugation has emerged as an effective strategy for improving peptide bioactivity, stability, and pharmacological performance. In the present study, TAT-TIL conjugates were rationally designed to enhance the biological functions of amphibian skin-derived Bowman–Birk-like trypsin inhibitor peptides. Our results demonstrate that conjugation with the cell-penetrating peptide TAT markedly enhances antimicrobial activity and modulates antiproliferative effects, while the introduction of a glycine linker further improves biological performance. These findings suggest that CPP-mediated modification, rather than protease inhibitory activity itself, plays a dominant role in enhancing peptide bioactivity.

The amphibian skin-derived peptides are named Bowman–Birk-like trypsin inhibitors (BBLTIs), and they exhibit a conserved precursor structure consisting of 65–70 amino acids. This structure includes a signal peptide at the N-terminus, which is highly conserved, then an acidic spacer, and a mature peptide domain, concluding with the C-terminus [41,42,43,44]. They share a highly conserved disulphide-bridged loop consisting of eleven residues (CWTP1SXPPXPC), which plays a pivotal role in their trypsin inhibitory activity [45,46]. Natural BBLTI peptides not only have a potent anti-trypsin ability but also possess multiple biological functions, including slight antimicrobial activity, weak anticancer activity, and the ability to reduce inflammation, interfere with the absorption of nutrients, and regulate the immune system [47,48,49]. However, their relatively modest biological potency restricts their therapeutic application. Therefore, conjugation with CPPs represents a promising approach to enhance peptide functional properties.

In this study, analogues were constructed by conjugating TAT with a trypsin inhibitory loop (TIL). OSTI-2734 was generated by directly fusing TAT with the TIL of OSTI-1872. In OSTI-2886, a diglycine (-GG-) linker was introduced between TAT and TIL to facilitate conjugation and improve structural flexibility. The final analogue, OSTI-2785, is derived from OSTI-2886 by disrupting the disulfide bridge, which is accomplished through the elimination of the last cysteine residue. The majority of reported protease inhibitor peptides typically demonstrate a singular inhibitory activity, targeting either trypsin or chymotrypsin [27,28], whereas there are still a limited number of peptides that have demonstrated dual inhibitory activity [26]. OSTI-1872 exhibits dual inhibitory activity, including potent inhibition of trypsin and a modest inhibitory effect on chymotrypsin, and this dual activity is preserved in all analogues. Notably, the enhanced chymotrypsin-inhibiting specificity observed in OSTI-2886 and OSTI-2785 is attributable to the substitution of the P1 lysine residue in the TIL with phenylalanine.

Regarding antimicrobial effectiveness, all TAT-conjugated analogues exhibited significantly enhanced antimicrobial activity against both Gram-negative bacteria and MRSA compared with the parent peptide OSTI-1872. This improvement is likely driven by increased electrostatic interactions between the positively charged TAT moiety and negatively charged bacterial membranes. Gram-negative bacterial membranes, particularly those of *E. coli*, possess a higher density of negatively charged phospholipids than Gram-positive bacteria; enhanced electrostatic attraction likely facilitates peptide binding and membrane association [50]. Notably, OSTI-2785, which lacks intact protease inhibitory activity due to disruption of the disulfide bridge, showed antimicrobial activity comparable to OSTI-2886. This observation indicates that protease inhibitory function is not essential for antimicrobial activity enhancement following TAT conjugation, suggesting that CPP-mediated membrane interaction or intracellular targeting may represent the primary mechanism of action.

All peptides exhibited low membrane permeabilization against three *E. coli* strains at the tested concentrations. OSTI-2734 showed negligible membrane permeability to the tested *E. coli* strains across all concentrations. Membrane permeability assays revealed that TAT-conjugated peptides induced minimal membrane disruption in Gram-negative bacteria, suggesting that their antimicrobial activity is unlikely to rely on direct membrane lysis. Instead, these peptides may exert antibacterial effects through non-lytic mechanisms, potentially involving intracellular targets following CPP-mediated translocation. Meanwhile, these peptides vary in their degrees of membrane-disruptive activity to different tested Gram-negative bacteria strains, but they were positively correlated with the concentration of peptides.

Consistent with this hypothesis, time-kill kinetic assays demonstrated relatively slow bactericidal activity at MICs but more rapid bacterial killing at higher peptide concentrations. These findings suggest a concentration-dependent mode of action that may involve progressive intracellular accumulation rather than immediate membrane destruction. The distinct bactericidal kinetics observed among different analogues further indicate that structural features such as linker incorporation may influence cellular uptake efficiency or intracellular activity.

Despite the substantial improvement in antimicrobial activity observed across all analogues, it is noteworthy that OSTI-2734 did not exhibit a detectable enhancement in antiproliferative activity, which aligns with findings from previous studies [28]. However, OSTI-2886 featuring the linker (“-GG-”), in particular, displayed both potent antimicrobial and significantly enhanced antiproliferative activity compared to OSTI-2734 and the parent peptide OSTI-1872, which strongly indicates the significance of the linker in improving the biological activity of modified peptides. The enhanced activity is likely attributable to the structural flexibility introduced by peptide linkers. Such flexibility can influence the overall peptide conformation, which affects binding affinity, receptor recognition, and cellular uptake, while enabling the linked segments to adopt favourable conformations for optimal interaction with biological targets. In addition, flexible linkers may facilitate adaptation to the target environment. By spatially separating functional domains within a peptide or peptide conjugate, linkers can reduce steric hindrance and interference, allowing each domain to function more effectively [51,52,53].

Among the designed analogues, OSTI-2886, which contains a di-glycine (-GG-) linker, exhibited the most pronounced enhancement in both antimicrobial and antiproliferative activities. The improved activity can be attributed to the structural flexibility introduced by the glycine linker, which can reduce steric hindrance between functional domains and facilitate more favourable conformational adaptation for target interaction. Flexible linkers may also spatially separate functional regions, thereby enabling independent activity of the CPP and inhibitory loop. Importantly, molecular dynamics simulation results support this interpretation. OSTI-2886 demonstrated increased membrane contact surface area and occasional deeper insertion of non-polar residues into the hydrophobic membrane region compared with other analogues. These findings suggest that linker-mediated structural flexibility may facilitate membrane interaction and cellular uptake, thereby contributing to enhanced biological activity. In addition to antimicrobial effects, TAT-TIL conjugates displayed differential antiproliferative activity against cancer cells. Notably, OSTI-2886 and OSTI-2785 exhibited significantly enhanced antiproliferative effects, whereas OSTI-2734 showed limited activity, further underscoring the importance of linker incorporation in activity optimization.

However, enhanced anticancer activity was accompanied by increased cytotoxicity toward normal HaCaT cells, as well as dose-dependent hemolytic effects, particularly for OSTI-2886. These findings indicate limited cellular selectivity, suggesting that TAT-mediated cellular uptake may occur in both normal and cancer cells. Such non-selective cytotoxicity represents a potential limitation for therapeutic application and highlights the need for further structural optimization to improve target specificity.

Apoptosis assays revealed that OSTI-2886 did not induce apoptosis in cancer cells, indicating that its antiproliferative effects may involve alternative mechanisms such as cell cycle arrest or metabolic disruption. Further studies are, therefore, warranted to elucidate the underlying intracellular pathways.

Molecular dynamics simulations revealed that the peptide–membrane association was predominantly driven by electrostatic interactions, as evidenced by substantially higher electrostatic interaction energies compared with van der Waals interactions. TAT-conjugated peptides exhibited increased contact surface area and more rapid equilibrium binding with the membrane, supporting their enhanced membrane affinity. Furthermore, centroid distance analysis indicated distinct membrane insertion behaviours among the analogues. OSTI-2734 is primarily associated with membrane headgroups, whereas OSTI-2886 occasionally penetrates into the hydrophobic membrane core. These differences in membrane interaction behaviour correlate with their respective biological activities, providing mechanistic insight into the enhanced efficacy of linker-containing conjugates.

Collectively, these findings demonstrate that CPP conjugation represents an effective strategy for enhancing the biological activity of protease inhibitor peptides. The results further highlight the importance of linker design in modulating peptide structure, membrane interaction, and biological function. This study provides a rational framework for the development of multifunctional peptide conjugates and supports further investigation of CPP-modified peptides as potential antimicrobial and anticancer agents.

## 5. Conclusions

In summary, conjugation of the TAT cell-penetrating peptide with the BBI-derived trypsin inhibitory loop (TIL) significantly enhanced the biological activities of the modified peptides, particularly their antimicrobial and antiproliferative effects. Notably, protease inhibitory activity does not appear to be an essential determinant of these enhanced functions, revealing a bioactivity enhancement mechanism largely driven by CPP-mediated modification. Furthermore, results obtained with OSTI-2886 demonstrate that the introduction of a glycine linker (-GG-) substantially promotes activity enhancement, establishing linker design as an important determinant of peptide bioactivity. Importantly, these findings indicate that TAT-TIL conjugates represent promising lead structures for further development due to their improved bioactivity and low haemolytic effects. However, the increased cytotoxicity observed in normal cells may limit their therapeutic translation and highlights the need for further optimization to improve selectivity and safety. In addition, the precise mechanisms underlying cellular uptake and intracellular activity of these conjugates remain to be elucidated.

Overall, this study supports CPP conjugation as an effective strategy for enhancing peptide function and provides a rational framework for the design of multifunctional peptide therapeutics, thereby facilitating the future development of peptide-based antimicrobial and anticancer agents.

## Figures and Tables

**Table 1:** The physicochemical properties of OSTI-1872 and its analogues.

Peptides	Sequences	Hydrophilicity	Net Charge
OSTI-1872	AALKGCWTKSIPPRPCF-NH_2_	−0.21	3.91
OSTI-2734	RKKRRQRRR-CWTKSIPPRPCK-NH_2_	1.23	11.91
OSTI-2886	RKKRRQRRR-GG-CWTFSIPPRPCF-NH_2_	0.65	9.91
OSTI-2785	RKKRRQRRR-GG-CWTFSIPPRPF-NH_2_	0.72	9.95

**Table 2:** Inhibitory activity of OSTI-1872 and its analogues against trypsin.

Peptides	Sequences	Ki (μM)	
		Trypsin	Chymotrypsin
OSTI-1872	AALKGCWTKSIPPRPCF-NH_2_	0.03491	174.3
OSTI-2734	RKKRRQRRR-CWTKSIPPRPCK-NH_2_	0.03658	282.8
OSTI-2886	RKKRRQRRR-GG-CWTFSIPPRPCF-NH_2_	9.326	0.2561
OSTI-2785	RKKRRQRRR-GG-CWTFSIPPRPF-NH_2_	16.38	0.5387

**Table 3:** MIC/MBC (μM) values of peptides against ten microorganisms.

Bacteria Strains		OSTI-1872	OSTI-2734	OSTI-2886	OSTI-2785
Gram-negative bacteria	*E. coli* (ATCC CRM 8739)	128/256	8/8	2/2	2/2
	*E. coli* (BAA 2340)	32/32	16/16	2/4	4/4
	*E. coli* (NCTC 13846)	32/32	16/16	2/4	2/4
	*P. aeruginosa* (ATCC CRM 9027)	256/512	16/16	16/16	16/16
	*K. pneumoniae* (ATCC CRM 43861)	>512	16/16	8/8	8/8
	*A. baumannii* (BAA 747)	>512	>512	32/64	32/64
Gram-positive bacteria	*S. aureus* (ATCC CRM 6538)	>512	>512	>512	>512
	*E. faecium* (NCTC 12697)	>512	>512	>512	>512
	MRSA (NCTC 12493)	>512	16/16	8/8	16/16
Fungus	*C. albicans* (ATCC 10231)	>512	>512	>512	>512

**Table 4:** The values of MIC/MBC against *E. coli* (ATCC CRM 8739) after peptides were incubated in a salt ion medium.

MIC/MBC (μM)				
Salts	OSTI-1872	OSTI-2734	OSTI-2886	OSTI-2785
1% DMSO	128/256	8/8	2/2	2/2
FeCl_3_	128/256	8/16	2/8	2/4
CaCl_2_	>512	16/16	8/8	4/4
NaCl	>512	4/4	1/1	2/4
KCl	128/256	8/16	2/4	2/4
MgCl_2_	>512	16/16	4/4	4/4
NH_4_Cl	128/256	8/16	2/4	2/2
Serum	128/128	8/16	2/4	2/4

**Table 5:** IC_50_ of peptides against human cancer cell lines.

Peptides	IC_50_ (μM)				
	H838	U251MG	MCF-7	HCT116	HaCaT
OSTI-1872	N.I.	N.I.	N.I.	N.I.	N.I.
OSTI-2734	N.I.	N.I.	N.I.	N.I.	906.1
OSTI-2886	31.90	21.99	58.90	17.19	25.01
OSTI-2785	8.779	28.80	3.611	21.32	29.54

## Data Availability

The data supporting the findings of this study are available within the article and its Supplementary Information Files.

## Supplementary Materials

The following supporting information can be downloaded at https://www.mdpi.com/article/10.3390/biom16040511/s1. Figure S1, MALDI-TOF MS spectrometry of the four synthetic peptides: (a) OSTI-1872, (b) OSTI-2734, (c) OSTI-2886, and (d) OSTI-2785; Table S1. Molecular weight (MW) of OSTI-1872 and analogues; Figure S2, Representative optical density measurements used for MIC determination of selected microorganisms.
