# Supplementary material for: Design of TAT-Conjugated Bowman–Birk Trypsin Inhibitor Peptides with Enhanced Antimicrobial and Antiproliferative Activities
Source: Biomolecules. 2026 Mar 30;16(4):511. doi: 10.3390/biom16040511 (PMC13112966; doi:10.3390/biom16040511)
Supplement: Supplementary file 1 [file biomolecules-16-00511-s001.zip › biomolecules-4194211-supplementary.pdf]

biomolecules

1. Supplementary data - MALDI-TOF MS spectrometry

OSTI-1872 and its three analogues were synthesized using a solid-phase peptide synthesizer, then, they were identified via MALDI-TOF Mass spectrometry (Figure S1) through the analysis of their molecular masses. The masses of the analogues were identical to the theoretical masses, indicating that the peptides of interest were successfully acquired (Table S1).

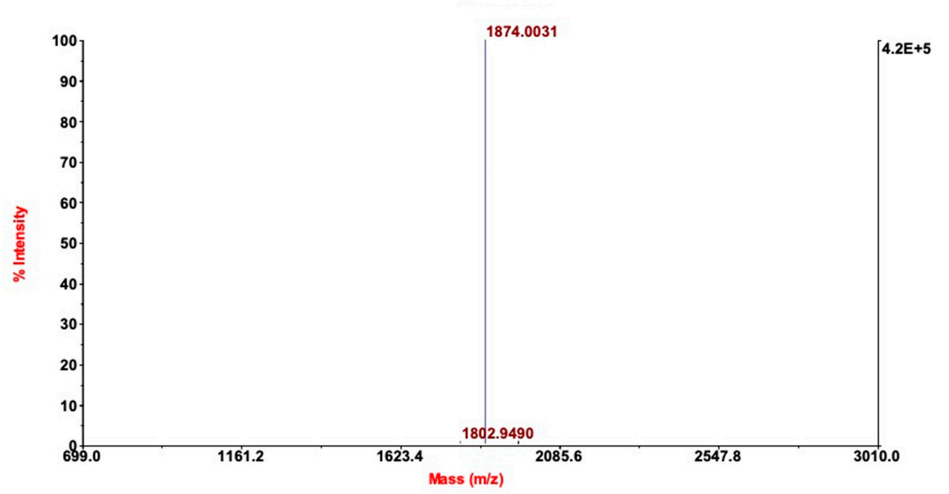

(a)

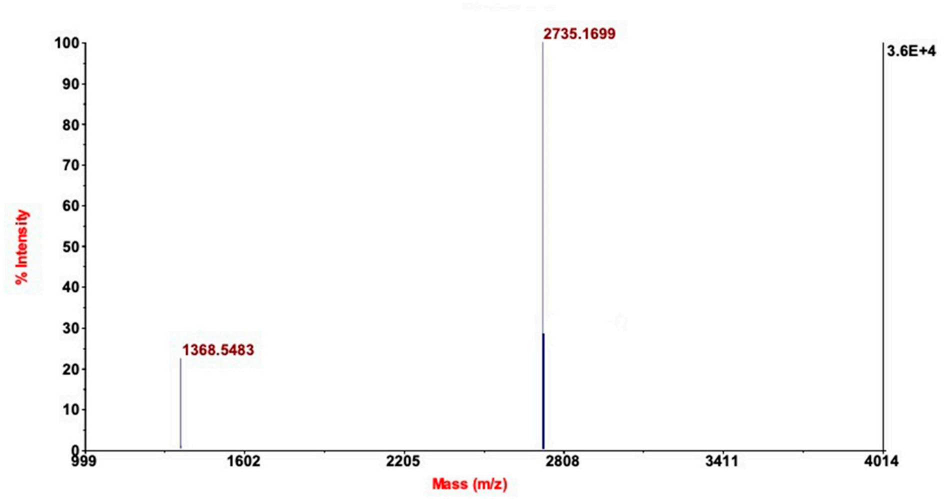

(b)

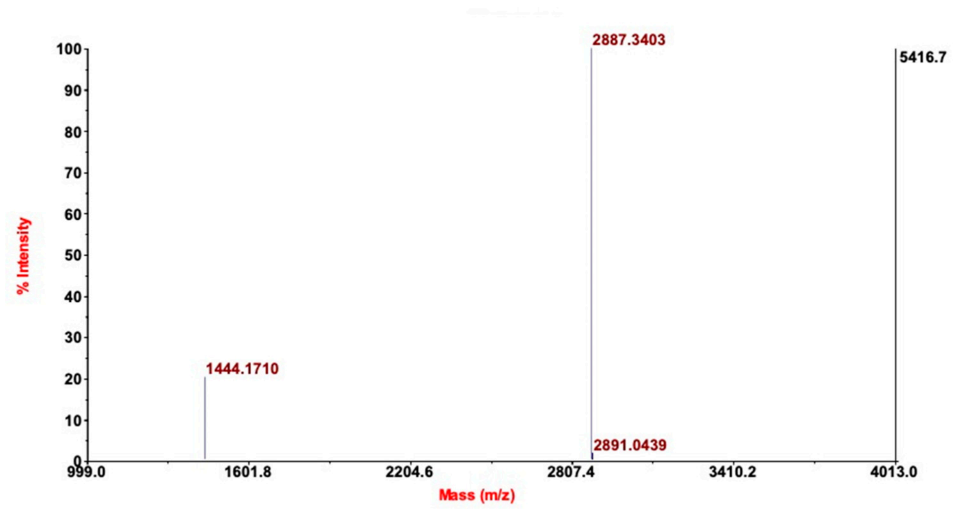

(c)

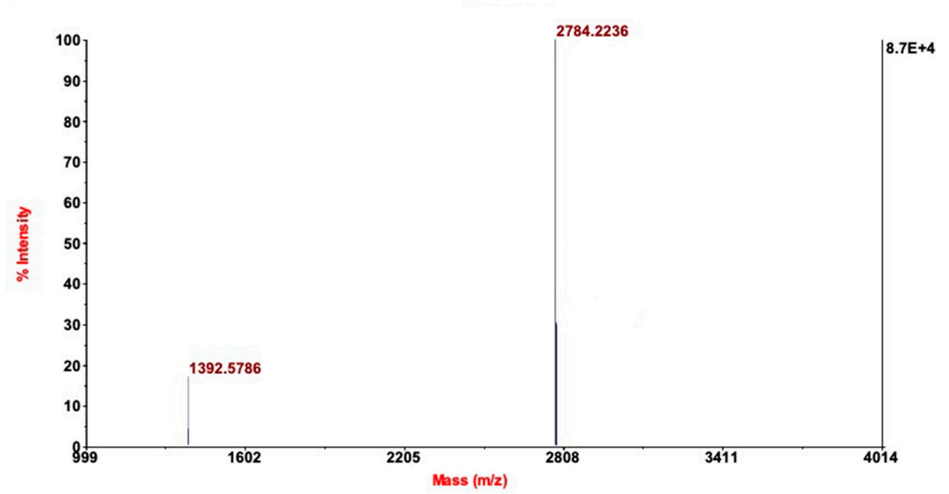

(d)

Figure S1. MALDI-TOF MS spectrometry of the four synthetic peptides: (a) OSTI-1872; (b) OSTI-2734; (c) OSTI-2886 and (d) OSTI-2785.

Table S1. Molecular weight (MW) of OSTI-1872 and analogues.

Peptides	Sequences	MW (m/z)
OSTI-1872	AALKGCWTKSIPPRPCF-NH_2	1872
OSTI-2734	RKKRRQRRR-CWTKSIPPRPCK-NH_2	2734
OSTI-2886	RKKRRQRRR-GG-CWTFSIPPRPCF-NH_2	2886
OSTI-2785	RKKRRQRRR-GG-CWTFSIPPRPF-NH_2	2785

2. Supplementary data – MIC/MBC assays

Figure S2. Representative optical density measurements used for MIC determination of selected microorganisms.

2.1. *E.coli* (ATCC CRM 8739)

1872-E.coli	G	512	256	128	64	32	16	8	4	2	1	B	
A	0.048	0.05	0.04	0.049	0.045	0.049	0.047	0.047	0.048	0.049	0.05	0.046	550
B	0.643	0.056	0.056	0.051	0.391	0.34	0.477	0.454	0.492	0.548	0.541	0.05	550
C	0.601	0.052	0.056	0.055	0.248	0.351	0.466	0.425	0.418	0.506	0.462	0.047	550
D	0.622	0.051	0.055	0.055	0.335	0.269	0.454	0.469	0.483	0.514	0.525	0.046	550
E	0.044	0.541	0.523	0.534	0.051	0.172	0.13	0.043	0.047	0.046	0.046	0.047	550
F	0.041	0.042	0.045	0.049	0.046	0.047	0.048	0.048	0.045	0.044	0.046	0.045	550
G	0.046	0.045	0.045	0.045	0.045	0.044	0.045	0.044	0.046	0.044	0.045	0.044	550
H	0.045	0.045	0.046	0.045	0.043	0.044	0.043	0.044	0.044	0.045	0.046	0.046	550
Growth	0.622	1%	1%	1%	60%	51%	75%	71%	77%	87%	86%	0.047667	
		1%	1%	1%	35%	53%	73%	66%	64%	80%	72%		
		1%	1%	1%	50%	39%	71%	73%	76%	81%	83%		
		1%	1%	1%	48%	47%	73%	70%	73%	83%	80%		

1872-E.coli	G	512	256	128	64	32	16	8	4	2	1	B	
A	0.045	0.047	0.041	0.042	0.041	0.041	0.039	0.04	0.04	0.044	0.041	0.039	550
B	0.505	0.056	0.061	0.062	0.1	0.426	0.402	0.329	0.362	0.446	0.464	0.044	550
C	0.538	0.052	0.061	0.059	0.086	0.424	0.359	0.334	0.36	0.42	0.482	0.048	550
D	0.557	0.052	0.054	0.056	0.103	0.443	0.38	0.381	0.414	0.452	0.527	0.041	550
DMSO	0.545	0.493	0.462	0.465	0.513	0.535	0.05	0.048	0.042	0.041	0.046	0.044	550
POSITIVE	0.045	0.041	0.046	0.045	0.043	0.045	0.045	0.044	0.044	0.043	0.045	0.044	550
G	0.046	0.046	0.046	0.047	0.048	0.045	0.046	0.044	0.044	0.043	0.044	0.043	550
H	0.045	0.045	0.046	0.047	0.045	0.047	0.043	0.045	0.043	0.044	0.045	0.048	550
	0.533333	2%	3%	4%	11%	78%	73%	58%	65%	82%	86%	0.044333	
		2%	3%	3%	9%	78%	64%	59%	65%	77%	90%		
		2%	2%	2%	12%	82%	69%	69%	76%	83%	99%		
		2%	3%	3%	11%	79%	69%	62%	68%	81%	91%		

1872-E.coli	G	512	256	128	64	32	16	8	4	2	1	B	
A	0.046	0.047	0.043	0.039	0.038	0.04	0.037	0.039	0.043	0.042	0.039	0.044	550
B	0.543	0.056	0.063	0.076	0.413	0.42	0.391	0.392	0.436	0.486	0.49	0.046	550
C	0.553	0.058	0.07	0.076	0.378	0.418	0.41	0.405	0.463	0.468	0.472	0.044	550
D	0.574	0.056	0.068	0.078	0.363	0.426	0.408	0.415	0.484	0.498	0.497	0.043	550
DMSO	0.633	0.592	0.545	0.052	0.051	0.049	0.051	0.051	0.051	0.05	0.05	0.04	550
F	0.044	0.045	0.043	0.045	0.044	0.046	0.045	0.043	0.046	0.043	0.044	0.044	550
G	0.045	0.048	0.046	0.045	0.045	0.044	0.045	0.045	0.044	0.044	0.045	0.045	550
H	0.045	0.047	0.045	0.045	0.043	0.044	0.043	0.043	0.044	0.044	0.045	0.045	550
	0.556667	2%	4%	6%	72%	73%	68%	68%	76%	86%	87%	0.044333	
		3%	5%	6%	65%	73%	71%	70%	82%	83%	83%		
		2%	5%	7%	62%	74%	71%	72%	86%	89%	88%		
		2%	4%	6%	66%	74%	70%	70%	81%	86%	86%		

	G	128	64	32	128	64	32	32	16	8	4	B	
E. coli		PURE 1891			PURE 1853			PURE 2734					
A	0.632	0.119	0.078	0.056	0.05	0.047	0.29	0.062	0.056	0.05	0.395	0.041	550
B	0.544	0.127	0.083	0.053	0.056	0.052	0.276	0.068	0.065	0.051	0.412	0.044	550
C	0.569	0.13	0.085	0.102	0.056	0.048	0.238	0.069	0.063	0.05	0.364	0.04	550
								DMSO	Norfloxacin	2	1	B	
										PURE 2734			
D	0.563	0.518	0.495	0.507	0.514	0.508	0.538	0.488	0.047	0.431	0.471	0.047	550
E	0.595	0.497	0.519	0.482	0.514	0.513	0.487	0.468	0.042	0.416	0.42	0.047	550
F	0.646	0.55	0.532	0.582	0.542	0.576	0.586	0.53	0.041	0.461	0.518	0.046	550
G	0.046	0.047	0.046	0.045	0.045	0.046	0.045	0.046	0.047	0.047	0.046	0.044	550
H	0.043	0.045	0.045	0.043	0.045	0.045	0.045	0.045	0.045	0.045	0.044	0.044	550
	0.581667											0.041667	
		14%	7%	3%	2%	1%	46%	4%	3%	2%	65%		
		16%	8%	2%	3%	2%	43%	5%	4%	2%	69%		
		16%	8%	11%	3%	1%	36%	5%	4%	2%	60%		
		15%	7%	5%	2%	1%	42%	5%	4%	2%	65%		
										72%	80%		
										69%	70%		
										78%	88%		
										73%	79%		

	G	512	256	128	64	32	16	8	4	2	1	B	
A	0.044	0.044	0.044	0.044	0.045	0.044	0.044	0.045	0.044	0.043	0.042	0.043	550
2734	0.742	0.163	0.072	0.071	0.065	0.059	0.049	0.045	0.381	0.491	0.6	0.644	550
	0.637	0.147	0.078	0.071	0.065	0.059	0.055	0.045	0.307	0.423	0.524	0.565	550
	0.633	0.187	0.073	0.076	0.069	0.063	0.058	0.052	0.298	0.418	0.502	0.556	550
1853	0.634	0.085	0.049	0.064	0.047	0.04	0.443	0.486	0.485	0.524	0.58	0.54	550
	0.625	0.083	0.082	0.064	0.049	0.04	0.44	0.485	0.494	0.53	0.582	0.517	550
	0.629	0.086	0.078	0.056	0.048	0.037	0.462	0.512	0.54	0.564	0.628	0.569	550
H	0.653	0.517	0.601	0.046	0.045	0.042	0.044	0.045	0.043	0.043	0.044	0.047	550
	DMSO												
	0. 670667											0. 044333	
		19%	4%	4%	3%	2%	1%	0%	54%	71%	89%		
		16%	5%	4%	3%	2%	2%	0%	42%	60%	77%		
		23%	5%	5%	4%	3%	2%	1%	41%	60%	73%		
		6%	1%	3%	0%	−1%	64%	71%	70%	77%	86%		
		6%	6%	3%	1%	−1%	63%	70%	72%	78%	86%		
		7%	5%	2%	1%	−1%	67%	75%	79%	83%	93%		
		19%	5%	5%	4%	3%	2%	0%	45%	64%	79%		
		6%	4%	3%	1%	−1%	65%	72%	74%	79%	88%		

E. coli	G	64	32	16	64	32	16	16	8	4	B	12	
A	0.043	0.045	0.042	0.042	0.043	0.045	0.044	0.045	0.045	0.044	0.043	0.045	550
		1891			1853			2734					
B	0.536	0.287	0.419	0.415	0.064	0.407	0.427	0.065	0.061	0.488	0.045	0.045	550
C	0.488	0.324	0.397	0.374	0.061	0.322	0.407	0.065	0.056	0.412	0.039	0.045	550
D	0.518	0.329	0.391	0.385	0.057	0.32	0.416	0.066	0.058	0.447	0.044	0.045	550
E	0.043	0.47	0.463	0.464	0.043	0.044	0.045	0.044	0.043	0.045	0.045	0.045	550
		DMSO											
F	0.043	0.07	0.064	0.049	0.045	0.044	0.045	0.045	0.044	0.044	0.045	0.043	550
		Norfloxacin											
G	0.046	0.045	0.045	0.045	0.044	0.044	0.045	0.044	0.045	0.045	0.043	0.045	550
H	0.044	0.048	0.046	0.043	0.043	0.044	0.043	0.044	0.043	0.043	0.044	0.046	550
	0. 514										0. 042667		
		52%	80%	79%	5%	77%	82%	5%	4%	94%			
		60%	75%	70%	4%	59%	77%	5%	3%	78%			
		61%	74%	73%	3%	59%	79%	5%	3%	86%			
		57%	76%	74%	4%	65%	79%	5%	3%	86%			

8739	G	16	8	4	2	1	16	8	4	2	1	B	
		2785					2382						
A	0.676	0.051	0.051	0.045	0.043	0.401	0.057	0.054	0.046	0.472	0.511	0.046	550
B	0.501	0.058	0.056	0.056	0.052	0.263	0.063	0.064	0.054	0.396	0.438	0.05	550
C	0.504	0.056	0.058	0.051	0.048	0.274	0.062	0.061	0.054	0.33	0.433	0.05	550
		16	8	4	2	1	64	32	16	8	4		
		2886					1859						
D	0.502	0.057	0.053	0.05	0.047	0.243	0.051	0.06	0.05	0.366	0.486	0.049	550
E	0.497	0.059	0.051	0.06	0.045	0.298	0.05	0.056	0.05	0.354	0.503	0.047	550
F	0.575	0.053	0.053	0.057	0.043	0.059	0.049	0.049	0.051	0.414	0.567	0.043	550
G	0.045	0.045	0.045	0.046	0.045	0.043	0.045	0.045	0.045	0.042	0.045	0.043	550
H	0.045	0.045	0.045	0.045	0.043	0.043	0.043	0.043	0.044	0.045	0.047	0.045	550
	0. 560333											0. 048667	
		0%	0%	−1%	−1%	69%	2%	1%	−1%	83%	90%		
		2%	1%	1%	1%	42%	3%	3%	1%	68%	76%		
		1%	2%	0%	0%	44%	3%	2%	1%	55%	75%		
		1%	1%	0%	0%	52%	2%	2%	1%	69%	81%		
		2%	1%	0%	0%	38%	0%	2%	0%	62%	85%		
		2%	0%	2%	−1%	49%	0%	1%	0%	60%	89%		
		1%	1%	2%	−1%	2%	0%	0%	0%	71%	101%		
		1%	1%	1%	−1%	30%	0%	1%	0%	64%	92%		

https://doi.org/10.3390/biom16040511

8739	G	128	64	32	16	8	4	8	4	64	32	B	
		2785						2886		1859			
A	0.521	0.053	0.049	0.05	0.048	0.055	0.051	0.051	0.048	0.05	0.048	0.046	550
B	0.439	0.056	0.056	0.057	0.059	0.066	0.054	0.057	0.052	0.055	0.05	0.048	550
C	0.446	0.054	0.055	0.056	0.057	0.063	0.052	0.054	0.051	0.053	0.052	0.046	550
	128	64	32	16	8	4	128	64	32	16	8	4	
	2382						1855						
D	0.075	0.074	0.075	0.076	0.062	0.046	0.108	0.106	0.082	0.051	0.392	0.47	550
E	0.065	0.073	0.066	0.072	0.052	0.046	0.131	0.131	0.085	0.088	0.39	0.442	550
F	0.067	0.068	0.068	0.069	0.057	0.049	0.209	0.122	0.087	0.067	0.436	0.505	550
G	0.046	0.046	0.044	0.046	0.045	0.044	0.045	0.044	0.045	0.042	0.045	0.044	550
H	0.044	0.044	0.045	0.046	0.044	0.045	0.042	0.044	0.043	0.045	0.045	0.045	550
	0. 468667											0. 046667	
		2%	1%	1%	0%	2%	1%	1%	0%	1%	0%		
		2%	2%	2%	3%	5%	2%	2%	1%	2%	1%		
		2%	2%	2%	2%	4%	1%	2%	1%	2%	1%		
		2%	2%	2%	2%	3%	1%	2%	1%	1%	1%		
	7%	6%	7%	7%	4%	0%	15%	14%	8%	1%	82%	100%	
	4%	6%	5%	6%	1%	0%	20%	20%	9%	10%	81%	94%	
	5%	5%	5%	5%	2%	1%	38%	18%	10%	5%	92%	109%	
	5%	6%	5%	6%	2%	0%	24%	17%	9%	5%	85%	101%	

	G	64	32	16	8	4	32	16	8	4	2	B	
		1855					2886						
A	0.495	0.093	0.087	0.414	0.356	0.376	0.054	0.062	0.058	0.049	0.404	0.045	550
B	0.395	0.106	0.092	0.3	0.347	0.372	0.061	0.074	0.06	0.052	0.051	0.05	550
C	0.395	0.094	0.092	0.352	0.32	0.347	0.061	0.079	0.063	0.054	0.052	0.05	550
	128	64	32	16	8	4	128	64	32	16	8	4	
	2785						2382						
D	0.054	0.055	0.052	0.051	0.077	0.048	0.073	0.071	0.074	0.068	0.057	0.046	550
E	0.048	0.054	0.049	0.052	0.074	0.048	0.07	0.071	0.071	0.072	0.053	0.049	550
F	0.048	0.054	0.052	0.052	0.074	0.047	0.071	0.069	0.069	0.073	0.052	0.044	550
	128	1855	128	64	1859	64	32	1859	32	16	1859	16	
G	0.124	0.124	0.123	0.056	0.058	0.055	0.05	0.047	0.046	0.356	0.314	0.508	550
	8	1859	8										
H	0.477	0.41	0.407	0.419	0.421	0.453	0.043	0.043	0.044	0.045	0.045	0.045	550
	0. 428333											0. 048333	
		12%	10%	96%	81%	86%	1%	4%	3%	0%	94%		
		15%	11%	66%	79%	85%	3%	7%	3%	1%	1%		
		12%	11%	80%	71%	79%	3%	8%	4%	1%	1%		
		13%	11%	81%	77%	83%	3%	6%	3%	1%	32%		
	1%	2%	1%	1%	8%	0%	6%	6%	7%	5%	2%	−1%	
	0%	1%	0%	1%	7%	0%	6%	6%	6%	6%	1%	0%	
	0%	1%	1%	1%	7%	0%	6%	5%	5%	6%	1%	−1%	
	0%	2%	1%	1%	7%	0%	6%	6%	6%	6%	1%	−1%	
	20%	20%	20%	2%	3%	2%	0%	0%	−1%	81%	70%	121%	
	113%	95%	94%	98%	98%	106%	−1%	−1%	−1%	−1%	−1%	−1%	

2.2. MRSA (NCTC 12493)

MRSA	G	512	256	128	64	128	64	32	16	256	128	64	
A	0.043	0.044	0.047	0.042	0.043	0.045	0.043	0.045	0.045	0.044	0.043	0.043	550
		1872				1891				1853			
B	0.381	0.258	0.443	0.445	0.401	0.08	0.123	0.29	0.368	0.081	0.063	0.164	550
C	0.381	0.274	0.373	0.395	0.371	0.087	0.148	0.252	0.345	0.072	0.066	0.424	550
D	0.393	0.218	0.385	0.375	0.359	0.085	0.137	0.235	0.356	0.068	0.064	0.059	550
	B	512	256	128	64	32	16	8					
		1920				2734							
E	0.043	0.089	0.182	0.296	0.305	0.071	0.063	0.315	0.044	0.045	0.045	0.044	550
F	0.048	0.085	0.164	0.293	0.318	0.074	0.064	0.137	0.043	0.045	0.046	0.046	550
G	0.048	0.078	0.165	0.313	0.338	0.071	0.063	0.326	0.045	0.043	0.043	0.044	550
		DMSO			Norfloxacin								
H	0.043	0.374	0.364	0.356	0.047	0.049	0.044	0.044	0.043	0.043	0.044	0.046	550
	0. 385												
	0. 046333												
		63%	117%	118%	105%	10%	23%	72%	95%	10%	5%	35%	
		67%	96%	103%	96%	12%	30%	61%	88%	8%	6%	112%	
		51%	100%	97%	92%	11%	27%	56%	91%	6%	5%	4%	
		60%	105%	106%	98%	11%	26%	63%	92%	8%	5%	50%	
		13%	40%	74%	76%	7%	5%	79%					
		11%	35%	73%	80%	8%	5%	27%					
		9%	35%	79%	86%	7%	5%	83%					
		11%	37%	75%	81%	8%	5%	63%					

https://doi.org/10.3390/biom16040511

MRSA	B	128	64	32	32	16	8	4	128	64	32	G	
		PURE 1891			PURE 2734				CRUDE 1840				
A	0.048	0.174	0.239	0.218	0.062	0.058	0.185	0.259	0.056	0.056	0.044	0.331	550
B	0.049	0.175	0.146	0.176	0.07	0.062	0.223	0.294	0.055	0.052	0.05	0.327	550
C	0.047	0.181	0.144	0.218	0.068	0.059	0.185	0.315	0.059	0.054	0.049	0.38	550
	DMSO	32	16	8	P								
		CRUDE 2369											
D	0.278	0.073	0.177	0.238	0.056	0.118	0.045	0.045	0.045	0.045	0.044	0.045	550
E	0.282	0.082	0.066	0.268	0.052	0.045	0.045	0.044	0.044	0.045	0.044	0.045	550
F	0.27	0.082	0.067	0.258	0.053	0.045	0.045	0.045	0.045	0.044	0.045	0.044	550
G	0.046	0.045	0.045	0.045	0.044	0.046	0.045	0.044	0.045	0.045	0.043	0.044	550
H	0.044	0.046	0.045	0.044	0.043	0.045	0.043	0.044	0.043	0.043	0.044	0.045	550
	0. 048											0. 346	
		42%	64%	57%	5%	3%	46%	71%	3%	3%	−1%		
		43%	33%	43%	7%	5%	59%	83%	2%	1%	1%		
		45%	32%	57%	7%	4%	46%	90%	4%	2%	0%		
		43%	43%	52%	6%	4%	50%	81%	3%	2%	0%		
		8%	43%	64%									
		11%	6%	74%									
		11%	6%	70%									
		10%	19%	69%									

MRSA	G	512	256	128	64	32	16	8	4	2	1	B	
A	0.044	0.045	0.044	0.055	0.046	0.045	0.044	0.044	0.044	0.046	0.044	0.023	550
2734	0.302	0.077	0.073	0.087	0.07	0.06	0.051	0.035	0.307	0.144	0.071	0.036	550
	0.348	0.071	0.067	0.074	0.073	0.063	0.056	0.436	0.445	0.411	0.252	0.034	550
	0.367	0.075	0.072	0.081	0.076	0.062	0.061	0.05	0.275	0.437	0.431	0.447	550
1853	0.365	0.085	0.072	0.108	0.367	0.378	0.464	0.299	0.258	0.372	0.35	0.112	550
	0.405	0.087	0.076	0.062	0.373	0.517	0.276	0.484	0.335	0.287	0.423	0.094	550
	0.412	0.079	0.07	0.118	0.372	0.324	0.408	0.333	0.466	0.297	0.309	0.19	550
H	0.39	0.416	0.43	0.039	0.038	0.038	0.045	0.045	0.043	0.043	0.044	0.045	550
	DMSO												
	0. 339											0. 035	
		14%	13%	17%	12%	8%	5%	0%	89%	36%	12%		
		12%	11%	13%	13%	9%	7%	132%	135%	124%	71%		
		13%	12%	15%	13%	9%	9%	5%	79%	132%	130%		
		16%	12%	24%	109%	113%	141%	87%	73%	111%	104%		
		17%	13%	9%	111%	159%	79%	148%	99%	83%	128%		
		14%	12%	27%	111%	95%	123%	98%	142%	86%	90%		
		13%	12%	15%	13%	9%	7%	46%	101%	97%	71%		
		16%	12%	20%	110%	122%	114%	111%	105%	93%	107%		

MRSA 12493	32	16	8	32	16	8	16	8	4	128	64	128	
	2785			2382			2886			1859		1855	
A	0.039	0.041	0.049	0.052	0.055	0.221	0.041	0.047	0.193	0.036	0.055	0.195	550
B	0.042	0.043	0.052	0.053	0.058	0.162	0.044	0.049	0.217	0.038	0.039	0.174	550
C	0.042	0.044	0.052	0.056	0.06	0.26	0.045	0.049	0.191	0.036	0.035	0.15	550
D	0.336	0.337	0.321	0.042	0.041	0.042	0.046	0.045	0.044	0.043	0.044	0.047	550
E	0.044	0.048	0.046	0.045	0.045	0.045	0.045	0.046	0.046	0.044	0.045	0.046	550
F	0.044	0.044	0.045	0.044	0.043	0.045	0.046	0.044	0.045	0.045	0.047	0.045	550
G	0.045	0.045	0.045	0.046	0.046	0.044	0.045	0.045	0.045	0.043	0.046	0.044	550
H	0.044	0.046	0.045	0.045	0.044	0.045	0.043	0.044	0.043	0.045	0.045	0.045	550

		2785			2886			2382					
	0. 331333												
	0. 041667												
550	−1%	0%	3%	4%	5%	62%	0%	2%	52%	−2%	5%	53%	
550	0%	0%	4%	4%	6%	42%	1%	3%	61%	−1%	−1%	46%	
550	0%	1%	4%	5%	6%	75%	1%	3%	52%	−2%	−2%	37%	
	0%	0%	3%	4%	6%	60%	1%	2%	55%	−2%	0%	45%	
	32	16	8	32	16	8	16	8	4	128	64	128	
550	2785			2382			2886			1859		1855	
550													
550													
550													
550													

MRSA	128	64	32	16	8	4	128	64	32	16	8	4	
	2886												
A	0.281	0.658	0.126	0.087	0.058	1.127	0.193	1.167	0.946	1.069	1.133	1.207	[illegible]
B	0.299	0.168	0.313	0.181	0.054	1.126	0.117	0.816	1.087	0.816	0.918	0.948	[illegible]
C	0.375	0.215	0.09	0.06	0.057	0.301	0.132	1.243	0.703	0.893	0.81	1.164	[illegible]
	128	64	32	16	8	4	128	64	32	16	8	4	
	2785												
D	0.389	0.206	0.125	0.091	0.038	0.055	0.78	0.77	0.682	0.72	0.692	0.952	[illegible]
E	0.362	0.849	0.126	0.1	0.062	0.055	0.336	0.875	0.868	0.611	0.745	0.966	[illegible]
F	0.282	0.258	0.217	0.39	0.05	0.063	0.77	0.417	0.59	0.885	0.827	0.937	[illegible]
G	0.045	0.046	0.045	0.047	0.047	0.045	1.091	1.059	1.107	0.033	0.032	0.03	[illegible]
H	0.044	0.045	0.045	0.046	0.045	0.044	0.044	0.045	0.044	0.045	0.045	0.046	[illegible]

	1. 085667											
	0. 031667											
[illegible]	24%	59%	9%	5%	2%	104%	15%	108%	87%	98%	104%	112%
[illegible]	25%	13%	27%	14%	2%	104%	8%	74%	100%	74%	84%	87%
[illegible]	33%	17%	6%	3%	2%	26%	10%	115%	64%	82%	74%	107%
	27%	30%	14%	7%	2%	78%	11%	99%	84%	85%	87%	102%
	128	64	32	16	8	4	128	64	32	16	8	4
[illegible]	34%	17%	9%	6%	1%	2%	71%	70%	62%	65%	63%	87%
[illegible]	31%	78%	9%	6%	3%	2%	29%	80%	79%	55%	68%	89%
[illegible]	24%	21%	18%	34%	2%	3%	70%	37%	53%	81%	75%	86%
[illegible]	30%	39%	12%	15%	2%	2%	57%	62%	65%	67%	69%	87%
[illegible]	128	64	32	16	8	4	128	64	32	16	8	4

MRSA 12493	G	64	32	16	8	4	64	32	16	8	4	B	
NB		P2785					P2382						
A	0.289	0.057	0.047	0.042	0.12	0.331	0.052	0.054	0.05	0.322	0.254	0.043	550
B	0.302	0.064	0.056	0.047	0.202	0.287	0.07	0.067	0.06	0.29	0.268	0.046	550
C	0.322	0.063	0.053	0.043	0.169	0.3	0.068	0.062	0.056	0.296	0.262	0.047	550
	32	16	8	4	128	64	32	16	128	64	32	16	
	P2886				P1859				P1855				
D	0.051	0.06	0.063	0.236	0.048	0.257	0.049	0.317	0.334	0.307	0.296	0.29	550
E	0.049	0.057	0.06	0.247	0.049	0.048	0.047	0.314	0.339	0.309	0.288	0.295	550
F	0.047	0.05	0.059	0.342	0.046	0.048	0.046	0.339	0.377	0.328	0.311	0.324	550
G	0.045	0.047	0.045	0.045	0.044	0.043	0.045	0.045	0.044	0.042	0.045	0.044	550
H	0.045	0.046	0.048	0.045	0.043	0.043	0.044	0.043	0.043	0.044	0.045	0.045	550
	0. 304333											0. 045333	
		7%	1%	−1%	41%	157%	4%	5%	3%	152%	115%		
		11%	6%	1%	86%	133%	14%	12%	9%	134%	122%		
		10%	5%	−1%	68%	140%	13%	10%	6%	138%	119%		
		9%	4%	0%	65%	143%	10%	9%	6%	141%	119%		
		9%	10%	105%	2%	116%	3%	149%	158%	144%	138%		
		7%	9%	111%	3%	2%	1%	147%	161%	145%	133%		
		3%	8%	163%	1%	2%	1%	161%	182%	155%	146%		
		6%	9%	126%	2%	40%	2%	152%	167%	148%	139%		
